# The Glucose-to-Lymphocyte Ratio Predicts All-cause Mortality and Cardiovascular Mortality in ST-Elevation Myocardial Infarction Patients: A Retrospective Study

**DOI:** 10.31083/RCM26065

**Published:** 2025-03-19

**Authors:** Jinfang Pu, Hongxing Zhang, Feng Wang, Yanji Zhou, Dajin Liu, Huawei Wang, Tao Shi, Sirui Yang, Fazhi Yang, Lixing Chen

**Affiliations:** ^1^The First Affiliated Hospital of Kunming Medical University, 650032 Kunming, Yunnan, China; ^2^Department of Pathogenic Biology and Immunology, Kunming Medical University, 650500 Kunming, Yunnan, China

**Keywords:** glucose-to-lymphocyte ratio, all-cause mortality, cardiovascular mortality, ST-elevation myocardial infarction, inflammation

## Abstract

**Background:**

Systemic inflammation and glucose metabolism are strongly associated with survival in ST-elevation myocardial infarction (STEMI) patients. Therefore, we aimed to assess whether the glucose-to-lymphocyte ratio (GLR) could be used to predict the prognosis of STEMI patients who received emergency percutaneous coronary intervention (PCI) treatment.

**Methods:**

The GLR was calculated as follows: GLR = glucose (mg/dL) / lymphocyte count (K/μL). Patients were divided into two groups according to the median GLR, with the low-GLR group (GLR <81) employed as the reference group. We used Cox proportional hazard regression analyses to determine the predictive value of clinical indicators. Kaplan‒Meier curves were used to plot survival curves for both groups. The receiver operating characteristic (ROC) curves were used to assess the predictive value of the GLR for the risk of all-cause mortality and cardiovascular mortality in STEMI patients. Meanwhile, to evaluate the predictive effectiveness of the models, we plotted the ROC curves for each model.

**Results:**

We retrospectively analyzed 1086 newly admitted patients with STEMI who underwent emergency PCI at the First Affiliated Hospital of Kunming Medical University from June 2018 to January 2023 (mean follow-up time, M ± standard deviation (SD): 1100.66 ± 539.76 days). The results showed that high GLR was associated with increased risks of all-cause mortality (hazard ratio (HR) = 2.530, 95% CI = 1.611–3.974, *p* < 0.001) and cardiovascular mortality (HR = 3.859, 95% CI = 2.225–6.691, *p* < 0.001). The optimal GLR threshold for predicting all-cause and cardiovascular death was 79.61 (K/μL), with a ROC for all-cause death of 0.678 (95% CI: 0.625–0.732, *p* < 0.001), a sensitivity of 77.4%, a specificity of 51.9%, and a ROC for cardiovascular death of 0.716 (95% CI: 0.666–0.767, *p* < 0.001), with a sensitivity of 88.4% and a specificity of 52.1%.

**Conclusions:**

The GLR may potentially predict all-cause mortality and cardiovascular mortality in STEMI patients who received emergency PCI treatment. A high GLR was associated with a greater risk of all-cause mortality and cardiovascular mortality in STEMI patients.

## 1. Introduction

ST-elevation myocardial infarction (STEMI) is a highly prevalent disease worldwide with high morbidity and mortality, and the global prevalence of STEMI is estimated to be 3.8% in people aged <60 years and 9.5% in people >60 years of age [1]. Percutaneous coronary intervention (PCI) is the mainstay of treatment for patients with STEMI. A meta-analysis in 2023 revealed that the overall mortality rate after PCI in patients with STEMI was 10% [2].

STEMI is one of the life-threatening coronary-related diseases and is the most common type of myocardial infarction. Moreover, the rupture or erosion of an atherosclerotic plaque precipitates STEMI. The rupture or erosion event activates the coagulation cascade and promotes platelet activation, culminating in the formation of an intraluminal thrombus and subsequent occlusion of the coronary artery. The resulting ischemia ultimately leads to myocardial necrosis [3]. Thus, improving myocardial ischemia and performing timely vascular recanalization are essential for treatment. PCI has become the preferred recanalization strategy for patients with STEMI. Although the mortality rate of STEMI has decreased, the mortality rate of STEMI is still higher than that of other diseases [4]. Therefore, identifying ways to predict the occurrence of adverse outcomes after emergency PCI in patients with STEMI is a major clinical problem.

A study has confirmed that inflammation plays an important role in the occurrence and development of STEMI and confirmed the role of lymphocytes in inflammation [5]. A study has also shown that hyperglycemia and inflammation are important prognostic factors in other acute coronary syndrome (ACS) conditions, such as takotsubo syndrome [6]. Lymphocytes are the strongest predictor of long-term death after acute myocardial infarction (AMI) [7]. Núñez J *et al*. [8] reported that a low lymphocyte count obtained within the first 96 hours after the onset of STEMI predicts the risk of recurrent myocardial infarction. Studies have also confirmed the relationship between glucose and inflammation and that stress hyperglycemia in patients with AMI amplifies the inflammatory immune response, which may lead to more extensive cardiac damage and worse cardiac functional outcomes [9]. However, in most previous studies, patient prognosis was usually assessed based on only lymphatic or glycemic aspects [7, 8, 9]. Recently, a new and readily available immune–metabolic marker, the glucose-to-lymphocyte ratio (GLR), has been used to assess the prognosis of several diseases. High GLRs are significantly associated with an increased risk of mortality from diseases such as T2 gallbladder cancer [10], type 2 diabetes mellitus and papillary thyroid cancer [11], nontraumatic cerebral hemorrhage [12], sepsis [13], pancreatic cancer [14], and acute severe pancreatitis [15], and provides specific prognostic value. However, no studies have confirmed the predictive value of the GLR for the prognosis of patients with STEMI; hence, this study aimed to investigate the prognostic impact of the GLR in STEMI patients who received emergency PCI treatment.

## 2. Materials and Methods

Study population: We included newly admitted patients diagnosed with STEMI who received emergency PCI at the First Affiliated Hospital of Kunming Medical University from June 2018 to January 2023. STEMI was defined according to the 2023 European Society of Cardiology (ESC) Guidelines for the Management of Acute Coronary Syndrome [16]. After admission, all patients were treated as the STEMI treatment guidelines recommended. The current study analyzed 1086 patients with STEMI who received emergency PCI treatment, excluding those who were lost to follow-up, those whose laboratory data were missing, or those who had a history of other serious diseases (e.g., autoimmune diseases, systemic diseases, malignant tumors, acute infection, and severe hepatorenal insufficiency).

Data collection: Demographic and clinical data and emergency electrocardiograms were collected upon patient admission. Demographic variables included age, sex, height, weight, and body mass index (BMI). Clinical data encompassed systolic blood pressure (SBP), diastolic blood pressure (DBP), Killip class for cardiac function, the Gensini score, and the global registry of acute coronary events (GRACE) risk score, as well as measurements of stent diameter and length, identification of the culprit artery, complications, and relevant medical history. Before the initiation of any therapeutic intervention, blood samples were drawn for routine laboratory tests, including blood cell analysis, myoglobin (Myo), creatine kinase-muscle/brain isoenzymes (CK-MB), and cardiac troponin I (cTnI). Following a 12-hour fasting period, additional blood samples were meticulously collected using standardized protocols and dispatched to the First Affiliated Hospital of Kunming Medical University laboratory for prompt analysis. The biochemical markers assessed included glucose, alanine aminotransferase (ALT), aspartate transaminase (AST), albumin (ALB), uric acid (UA), serum creatinine (Cr), total cholesterol (TC), low-density lipoprotein cholesterol (LDL-C), triglycerides (TGs), high-density lipoprotein cholesterol (HDL-C), and fibrinogen (Fib). The estimated glomerular filtration rate (eGFR) was calculated using the Cockcroft-Gault (CG) formula: for males, eGFR = (140 – age) × weight (kg) × 1.23/serum creatinine (mg/dL); for females, eGFR = (140 – age) × weight (kg) × 1.03/serum creatinine (mg/dL). The formula for GLR calculation was as follows: GLR = glucose (mg/dL) ÷ lymphocyte count (K/µL) [17].

Outcome and follow-up: All participants were followed up by trained research personnel who interviewed hospital patients, telephoned patients or their relatives, or checked their medical records. Investigators collected survival data via telephone interviews with patients or their families. The primary endpoints of this study were all-cause mortality and cardiovascular death. Cardiovascular death was defined as death from any cardiovascular cause: sudden cardiac death, death from arrhythmia, heart failure, myocardial infarction, or other cardiac causes, and all-cause mortality included death from all causes.

Statistical analysis: Measurement data conforming to a normal distribution are expressed as the mean ± standard deviation (mean ± SD), with comparisons between two groups conducted using the *t*-test. Skewed distributed data are presented as the median and quartiles (M (Q1, Q3)), and the Wilcoxon rank sum test was used for comparisons. Categorical data are described using frequency with composition ratios (N (%)), with the chi-square test or Fisher’s exact test utilized for comparative analysis. A univariate Cox proportional hazard model was used for covariate screening. Univariate and multivariate Cox proportional hazards models were established, and multivariate analyses were performed on variables with *p*-values < 0.05 for univariate analysis. The relationships between the GLR and all-cause mortality and cardiovascular mortality in STEMI patients treated with emergency PCI were explored.

To evaluate the association between the GLR and survival probability in STEMI patients treated with emergency PCI, we employed the Kaplan–Meier (KM) curve. Hazard ratios (HRs) and confidence intervals (CIs) were used as the evaluation indices. A *p*-value < 0.05 indicated a significant difference. Receiver operating characteristic (ROC) analysis was used to estimate the predictive value of the GLR for the risk of all-cause mortality and cardiovascular death in STEMI patients. Meanwhile, to assess the predictive effectiveness of the models, we plotted the ROC curves for each model. The data were analyzed statistically using SPSS ver. 27.0 (IBM SPSS Statistics 27.0.1, China). A two-sided *p*-value < 0.05 was considered indicative of statistical significance.

## 3. Results

### 3.1 Population and Patient Characteristics

This study included a total of 1086 newly admitted patients diagnosed with STEMI who underwent emergency PCI at the First Affiliated Hospital of Kunming Medical University from June 2018 to January 2023 (mean follow-up time, M ± SD: 1100.66 ± 539.76 days). In total, 106 patients died from all causes, and 90 patients died from cardiovascular disease. The age of all participants was 60.62 ± 12.02 years, and approximately 84.8% were male. Compared with those in the low-GLR group, patients in the high-GLR group had the following characteristics: they were older, had higher Gensini scores, had higher GRACE scores, had longer mean diameters and total lengths of stents, had higher Killip grades, received fewer angiotensin-converting enzyme inhibitors (ACEIs)/angiotonin receptor blocker (ARB)/angiotensin receptor–neprilysin inhibitors (ARNIs) and statin treatments; had higher CK-MB, Myo, cTnI, glucose, ALT, and high-density lipoprotein (HDL) levels; had lower red blood cell (RBC), hemoglobin (HB), lymphocyte, platelet (PLT), and TG counts; had more comorbidities, including prior diabetes and heart failure (*p* < 0.05). Data are presented in Table 1.

**Table 1.** **Baseline characteristics according to GLR**.

Variables		Total (n = 1086)	Low GLR (n = 543)	High GLR (n = 543)	*p*-value
	Age (years)	60.62 ± 12.02	59.1 ± 11.85	62.14 ± 12.00	<0.001
	Male, n (%)	921 (84.80)	449 (82.70)	472 (86.90)	0.052
	SBP (mmHg)	126.84 ± 23.63	126.41 ± 23.531	127.26 ± 23.734	0.557
	DBP (mmHg)	81.45 ± 16.134	81.24 ± 15.848	81.67 ± 16.427	0.663
	BMI (kg/m^2^)	24.29 ± 3.20	24.38 ± 3.05	24.20 ± 3.33	0.337
	Smokers, n (%)	617 (56.80)	337 (62.10)	280 (51.60)	<0.001
	Alcohol abuse, n (%)	217 (20.00)	104 (19.20)	113 (20.80)	0.495
Culprit artery					
	RCA, n (%)	419 (38.6)	203 (37.4)	216 (39.8)	0.418
	LM, n (%)	6 (0.6)	2 (0.2)	4 (0.6)	0.687
	LAD, n (%)	553 (50.9)	285 (52.5)	268 (49.4)	0.302
	LCX, n (%)	105 (9.7)	52 (9.6)	53 (9.8)	0.918
	Multivessel disease, n (%)	916 (84.7)	447 (82.5)	469 (86.9)	0.046
	The average length of the stent (mm)	23 (18, 28)	23 (18, 28)	24 (19, 28.5)	0.010
	The average diameter of the stent (mm)	3.01 ± 1.33	2.93 ± 1.34	3.08 ± 1.33	0.704
	The total length of the stent (mm)	28 (20, 42)	28 (19, 41)	29 (20, 46)	0.024
	Killip class >2, n (%)	364 (33.5)	157 (28.9)	207 (38.1)	<0.001
Comorbidities and medical history					
	Hypertension, n (%)	607 (55.9)	303 (55.8)	304 (56)	0.951
	Diabetes, n (%)	349 (32.1)	96 (17.7)	253 (46.6)	<0.001
	Hyperlipidemia, n (%)	347 (32)	169 (31.1)	178 (32.8)	0.558
	Prior stroke, n (%)	51 (4.7)	25 (4.6)	26 (4.8)	0.886
	Heart failure, n (%)	291 (26.8)	126 (23.2)	165 (30.4)	0.008
	Gensini risk score	64 (42, 89)	60 (40, 87)	68 (44, 92)	0.004
	GRACE score	151.87 ± 33.74	145.74 ± 30.80	158 ± 35.43	<0.001
Medication use					
	ACEI/ARB/ARNI, n (%)	479 (44.1)	258 (47.5)	221 (40.7)	0.024
	Aspirin, n (%)	1060 (97.6)	534 (98.3)	526 (96.9)	0.112
	P2Y12, n (%)	1057 (97.3)	532 (98)	525 (96.7)	0.188
	Beta-blockers, n (%)	772 (71.1)	398 (73.3)	374 (68.9)	0.108
	Statins, n (%)	1061 (97.7)	536 (98.7)	525 (96.7)	0.026
Laboratory parameters					
	CK-MB, ng/mL	18.62 (3.8, 70.45)	15.2 (2.20, 60.48)	23.8 (5.12, 80)	<0.001
	Myo, ng/mL	156.95 (58.22, 370.7)	111.92 (42.24, 321.72)	205 (82.79, 400)	<0.001
	cTnI, ng/mL	2.23 (0.12, 14.68)	1.52 (0.1, 12.348)	2.57 (0.16, 18.66)	0.013
	WBC (10^9^/L)	10.85 ± 3.62	10.77 ± 3.6	10.93 ± 3.64	0.470
	RBC (10^12^/L)	4.92 ± 0.71	4.97 ± 0.67	4.86 ± 0.74	0.010
	HB (g/L)	155 (141, 166)	156 (144, 166)	152 (139, 167)	0.012
	PLT (10^9^/L)	224.65 ± 71.68	231.04 ± 68.33	218.26 ± 74.39	0.003
	Lymphocyte (10^9^/L)	1.54 (1.13, 2.02)	1.91 (1.56, 2.43)	1.16 (0.87, 1.5)	<0.001
	Glucose (mmol/L)	6.39 (5.085, 8.64)	5.35 (4.65, 6.4)	8.19 (6.38, 11.53)	<0.001
	ALT (U/L)	42.2 (29, 63)	41.5 (30, 59)	43.1 (29, 66)	0.417
	AST (U/L)	73.25 (31.83, 174)	61 (31, 148.4)	83 (35, 210)	<0.001
	ALB (g/L)	39.52 ± 4.75	39.58 ± 4.58	39.47 ± 4.92	0.690
	Cr (mmol/L)	89.8 (76.2, 104.05)	88.3 (76, 101.9)	90.7 (76.7,106.8)	0.126
	UA (µmol/L)	386.25 (319.28, 466.85)	393 (328.6, 466.1)	371.7 (308, 467.6)	0.063
	eGFR (mL/min ×1.73 m^2^)	72.26 (55.50, 91.03)	75.42 (57.87, 93.81)	68.78 (52.36, 87.35)	<0.001
	TG (mmol/L)	1.49 (1.04, 2.06)	1.54 (1.11, 2.11)	1.43 (1.01, 2.00)	0.015
	TC (mmol/L)	4.49 ± 1.17	4.42 ± 1.11	4.55 ± 1.23	0.616
	HDL (mmol/L)	1.04 (0.88, 1.21)	1.01 (0.86, 1.18)	1.07 (0.90, 1.24)	<0.001
	LDL-C (mmol/L)	2.81 (2.19, 3.49)	2.76 (2.19, 3.41)	2.83 (2.19, 3.62)	0.178
	Fib	3.20 (2.66, 4.12)	3.2 (2.67, 3.99)	3.22 (2.66, 4.34)	0.469
	GLR level	80.82 (54, 123.91)	54 (41.4, 65.71)	123.89 (96, 174.41)	<0.001

The patients were divided into two groups according to the median GLR: the low group, GLR <81, and the high group, GLR ≥81, with the low GLR group used as the reference group. Continuous normally distributed variables are presented as the mean ± standard deviation, while non-normally distributed variables are reported as the median and interquartile range. Categorical variables are described using counts and percentages. One-way ANOVA and Kruskal–Wallis tests were employed. The χ^2^ test was utilized to evaluate differences in categorical variables between groups. The *p*-values were calculated to compare low and high GLR groups, with a significance threshold set at *p* < 0.05. GLR, glucose-to-lymphocyte ratio; SBP, systolic blood pressure; DBP, diastolic blood pressure; BMI, body mass index; RCA, right coronary artery; LCX, left circumflex artery; LAD, left anterior descending coronary artery; LM, left main coronary artery; ACEI/ARB/ARNI, angiotensin-converting enzyme inhibitors/angiotonin receptor blocker/angiotensin receptor–neprilysin inhibitor; Myo, myoglobin; CK-MB, creatine kinase-muscle/brain isoenzymes; cTnI, cardiac troponin I; WBC, white blood cell; PLT, platelet; RBC, red blood cell count; HB, hemoglobin; ALT, alanine aminotransferase; AST, aspartate aminotransferase; ALB, albumin; Cr, serum creatinine; UA, uric acid; eGFR, estimated glomerular filtration rate; TG, triglyceride; TC, total cholesterol; HDL, high-density lipoprotein; LDL-C, low-density lipoprotein; Fib, fibrinogen; GRACE, global registry of acute coronary events; ANOVA, analysis of variance.

### 3.2 Associations between the GLR and Endpoints

**Fig. 1.**
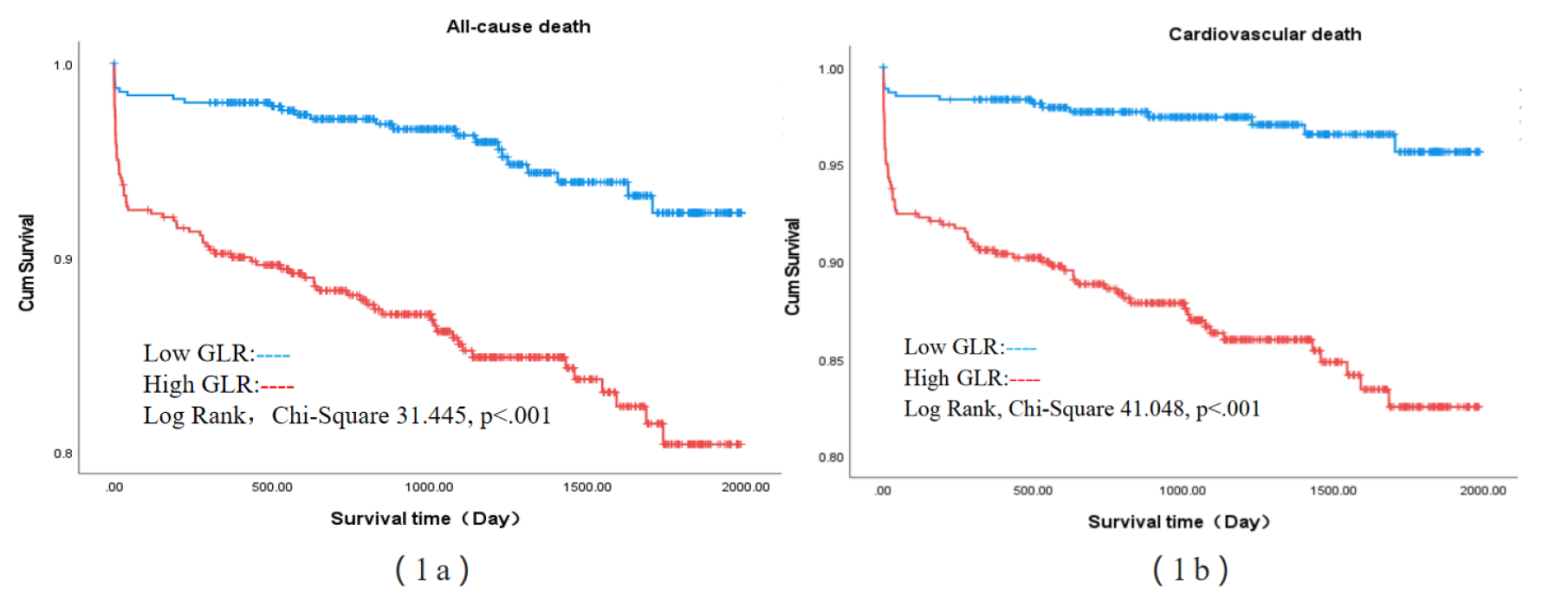
**(1a) (all-cause death) and (1b) (cardiovascular death): Kaplan‒Meier survival curves according to the median GLR for STEMI patients**. Low GLR: GLR <81; high GLR: GLR ≥81. STEMI, ST-elevation myocardial infarction; GLR, glucose-to-lymphocyte ratio.

Kaplan‒Meier analysis revealed that the cumulative incidence rates of all-cause death (log-rank, χ^2^ 31.445, *p* < 0.001) and cardiovascular death (log-rank, χ^2^ 41.048, *p* < 0.001) were significantly greater in STEMI patients in the high-GLR group than those in the low-GLR group (Fig. 1a,b).

### 3.3 A High GLR is an Independent Predictor of All-cause Mortality and Cardiovascular Mortality

We used the median value to divide the GLR into high and low groups, with the low group employed as the reference. Univariate analyses revealed that female sex, age, hypertension, diabetes, prior stroke, heart failure, the Gensini score, the GRACE score, the average diameter of the stent, the total length of the stent, Killip class ≥2, HB, Cr, ALB, UA, eGFR, Fib, and high GLR were common prognostic factors for all-cause mortality and cardiovascular death in STEMI patients.

The multivariate Cox proportional hazards analyses of all-cause death indicated that age (HR = 1.041, 95% CI = 1.012–1.070, *p* = 0.005), GRACE score (HR = 1.012, 95% CI = 1.002–1.021, *p* = 0.014), average diameter of the stent (HR = 0.682, 95% CI = 0.550–0.845, *p* < 0.001), UA level (HR = 1.003, 95% CI = 1.001–1.004, *p* < 0.001), and high GLR (HR = 2.179, 95% CI = 1.360–3.492, *p* < 0.001) were independent prognostic factors for all-cause death, as presented in Table 2.

**Table 2.** **Univariate and multivariate Cox analyses of variables associated with all-cause death**.

Variables	Unadjusted HR (95% CI)	*p*-value	Adjusted HR (95% CI)	*p*-value
Low GLR	Reference			
High GLR	3.298 (2.118–5.134)	<0.001	2.179 (1.360–3.492)	0.001
Age (years)	1.085 (1.066–1.105)	<0.001	1.041 (1.012–1.070)	0.005
Female	1.960 (1.259–3.052)	0.003	1.208 (0.724–2.015)	0.469
BMI (kg/m^2^)	0.949 (0.892–1.010)	0.100		
Alcohol abuse	1.236 (0.789–1.936)	0.354		
Hypertension	1.956 (1.287–2.973)	0.002	1.209 (0.775–1.886)	0.402
Diabetes	1.583 (1.076–2.331)	0.020	1.286 (0.842–1.964)	0.245
Prior stroke	2.753 (1.509–5.020)	<0.001	1.458 (0.787–2.701)	0.231
Heart failure	3.248 (2.218–4.757)	<0.001	1.267 (0.809–1.984)	0.301
Gensini score	1.008 (1.004–1.012)	<0.001	1.004 (0.999–1.009)	0.085
GRACE risk score	1.025 (1.020–1.030)	<0.001	1.012 (1.002–1.021)	0.014
RCA	0.901 (0.606–1.340)	0.606		
LM	2.406 (0.335–17.283)	0.383		
LAD	1.157 (0.789–1.697)	0.454		
LCX	0.739 (0.359–1.519)	0.410		
Multivessel disease	2.043 (1.032–4.405)	0.040	1.329 (0.645–2.737)	0.441
The average length of the stent (mm)	0.983 (0.963–1.004)	0.121		
The average diameter of the stent (mm)	0.752 (0.635–0.891)	<0.001	0.682 (0.550–0.845)	<0.001
The total length of the stent (mm)	1.010 (1.000–1.017)	0.043	1.008 (0.999–1.018)	0.071
Killip class ≥2	3.321 (2.252–4.898)	<0.001	1.019 (0.592–1.754)	0.946
CK-MB, ng/mL	1.000 (0.999–1.001)	0.932		
Myo, ng/mL	1.001 (1.000–1.000)	0.595		
cTnI, ng/mL	0.999 (0.994–1.005)	0.772		
WBC (10^9^/L)	1.038 (0.987–1.092)	0.150		
RBC (10^12^/L)	0.809 (0.632–1.036)	0.094		
HB (g/L)	0.991 (0.983–0.999)	0.024	1.003 (0.994–1.012)	0.497
PLT (10^9^/L)	1.001 (0.998–1.003)	0.715		
ALT (U/L)	1.000 (0.997–1.004)	0.898		
AST (U/L)	1.001 (1.000–1.002)	0.110		
ALB (g/L)	0.944 (0.914–0.975)	<0.001	0.998 (0.957–1.040)	0.915
Cr (mmol/L)	1.002 (1.001–1.004)	0.005	1.000 (0.997–1.003)	0.970
UA (µmol/L)	1.004 (1.003–1.006)	<0.001	1.003 (1.001–1.004)	<0.001
eGFR (mL/min ×1.73 m^2^)	0.965 (0.957–0.974)	<0.001	0.993 (0.978–1.008)	0.342
TGs (mmol/L)	0.873 (0.723–1.054)	0.158		
TC (mmol/L)	0.893 (0.755–1.056)	0.185		
HDL (mmol/L)	1.211 (0.740–1.982)	0.446		
LDL-C (mmol/L)	0.860 (0.706–1.047)	0.133		
Fib	1.090 (1.016–1.170)	0.016	0.989 (0.866–1.130)	0.875

The low GLR group (GLR <81) was used as the reference group. Adjusted for: glucose-to-lymphocyte ratio (GLR), age, body mass index (BMI), gender, alcohol abuse, hypertension, diabetes, prior stroke, heart failure, Gensini score, global registry of acute coronary events (GRACE) risk score, right coronary artery (RCA), left main coronary artery (LM), left circumflex artery (LCX), left anterior descending coronary artery (LAD), multivessel disease, the average length of the stent, the average diameter of the stent, the total length of the stent, the Killip classification ≥2, creatine kinase-muscle/brain isoenzymes (CK-MB), myoglobin (Myo), cardiac troponin I (cTnI), white blood cell (WBC) count, hemoglobin (HB), red blood cell (RBC) count, platelet (PLT), alanine aminotransferase (ALT), aspartate aminotransferase (AST), albumin (ALB), serum creatinine (Cr), uric acid (UA), estimated glomerular filtration rate (eGFR), triglycerides (TGs), high-density lipoprotein (HDL), total cholesterol (TC), low-density lipoprotein (LDL-C), and fibrinogen (Fib).

The multivariate Cox proportional hazards analysis revealed several independent prognostic factors for cardiovascular death. Specifically, the Gensini score (HR = 1.006, 95% CI = 1.001–1.011, *p* = 0.019), GRACE score (HR = 1.014, 95% CI = 1.004–1.024, *p* = 0.007), average diameter of the stent (HR = 0.593, 95% CI = 0.471–0.746, *p* < 0.001), total length of the stent (HR = 1.013, 95% CI = 1.003–1.022, *p* = 0.010), UA (HR = 1.003, 95% CI = 1.001–1.005, *p* < 0.001), and high GLR (HR = 3.348, 95% CI = 1.894–5.919, *p* < 0.001), as indicated in Table 3.

**Table 3.** **Univariate and multivariate Cox analyses of variables associated with cardiovascular death**.

Variables	Unadjusted HR (95% CI)	*p*-value	Adjusted HR (95% CI)	*p*-value
Low GLR	Reference			
High GLR	4.913 (2.862–8.435)	<0.001	3.348 (1.894–5.919)	<0.001
Age (years)	1.076 (1.056–1.097)	<0.001	1.024 (0.994–1.055)	0.120
Female	2.159 (1.353, 3.446)	<0.001	1.411 (0.814–2.447)	0.220
BMI (kg/m^2^)	0.969 (0.906–1.036)	0.354		
Alcohol abuse	1.300 (0.804–2.103)	0.285		
Hypertension	2.102 (1.325–3.334)	0.002	1.366 (0.837–2.227)	0.212
Diabetes	1.764 (1.164–2.674)	0.007	1.321 (0.837–2.085)	0.231
Prior stroke	2.985 (1.588–5.609)	<0.001	1.556 (0.810–2.987)	0.184
Heart failure	3.358 (2.219–5.083)	<0.001	1.171 (0.717–1.913)	0.527
Gensini score	1.010 (1.006–1.014)	<0.001	1.006 (1.001–1.011)	0.019
GRACE risk score	1.025 (1.020–1.030)	<0.001	1.014 (1.004–1.024)	0.007
RCA	0.978 (0.639–1.498)	0.919		
LM	2.614 (0.363–18.795)	0.340		
LAD	1.095 (0.723, 1.656)	0.669		
LCX	0.648 (0.283–1.484)	0.305		
Multivessel disease	2.641 (1.154, 6.047)	<0.001	1.682 (0.694–4.076)	0.250
The average length of the stent (mm)	0.980 (0.958–1.002)	0.078		
The average diameter of the stent (mm)	0.709 (0.595–0.846)	<0.001	0.593 (0.471–0.746)	<0.001
The total length of the stent (mm)	1.011 (1.003–1.020)	0.010	1.013 (1.003–1.022)	0.010
Killip class ≥2	3.659 (2.388–5.607)	<0.001	0.999 (0.546–1.826)	0.997
CK-MB, ng/mL	1.000 (0.996–1.004)	0.904		
Myo, ng/mL	1.000 (1.000–1.001)	0.232		
cTnI, ng/mL	0.999 (0.992–1.006)	0.779		
WBC (10^9^/L)	1.046 (0.990–1.104)	0.109		
RBC (10^12^/L)	0.804 (0.615–1.05)	0.109		
HB (g/L)	0.990 (0.981–0.999)	0.029	1.003 (0.944–1.013)	0.467
PLT (10^9^/L)	1.000 (0.997–1.003)	0.894		
ALT (U/L)	1.001 (0.998–1.004)	0.519		
AST (U/L)	1.001 (1.000–1.002)	0.055		
ALB (g/L)	0.943 (0.911–0.976)	<0.001	0.993 (0.949–1.038)	0.754
Cr (mmol/L)	1.002 (1.000–1.004)	0.014	1.000 (0.996–1.003)	0.833
UA (µmol/L)	1.005 (1.003–1.006)	<0.001	1.003 (1.001–1.005)	<0.001
eGFR (mL/min ×1.73 m^2^)	0.968 (0.959–0.976)	<0.001	0.994 (0.978–1.009)	0.427
TGs (mmol/L)	0.901 (0.745–1.090)	0.282		
TC (mmol/L)	0.908 (0.758–1.088)	0.295		
HDL (mmol/L)	1.229 (0.72–2.099)	0.449		
LDL-C (mmol/L)	0.880 (0.711–1.088)	0.238		
Fib	1.101 (1.029–1.179)	0.005	1.017 (0.888–1.165)	0.809

The low GLR group (GLR <81) was used as the reference group. Adjusted for: glucose-to-lymphocyte ratio (GLR), age, body mass index (BMI), gender, alcohol abuse, hypertension, diabetes, prior stroke, heart failure, Gensini score, global registry of acute coronary events (GRACE) risk score, right coronary artery (RCA), left circumflex artery (LCX), left main coronary artery (LM), left anterior descending coronary artery (LAD), multivessel disease, the average length of the stent, the average diameter of the stent, the total length of the stent, the Killip classification ≥2, creatine kinase-muscle/brain isoenzymes (CK-MB), myoglobin (Myo), cardiac troponin I (cTnI), white blood cell (WBC) count, hemoglobin (HB), red blood cell (RBC) count, platelet (PLT), alanine aminotransferase (ALT), aspartate aminotransferase (AST), albumin (ALB), serum creatinine (Cr), uric acid (UA), estimated glomerular filtration rate (eGFR), triglycerides (TGs), high-density lipoprotein (HDL), total cholesterol (TC), low-density lipoprotein (LDL-C), and fibrinogen (Fib). HR, hazard ratio; GRACE, global registry of acute coronary events.

Five adjusted models were used for the multivariate analysis. In Model 5, we adjusted for age, sex, multivessel disease status, the total length of the stent, average diameter of the stent, Killip classification, eGFR, and fibrinogen. Compared with the low-GLR group, all-cause mortality increased by 1.530-fold (HR = 2.530, 95% CI = 1.611–3.974, *p* < 0.001), and cardiovascular death increased by 2.859-fold (HR = 3.859, 95% CI = 2.225–6.691, *p* < 0.001) in the high-GLR group. The data are presented in Table 4.

**Table 4.** **Cox proportional hazard models for the association of GLR and the clinical outcomes**.

	All-cause death		Cardiovascular death	
Model	HR (95% CI)	*p*-value	HR (95% CI)	*p*-value
Unadjusted	3.298 (2.118–5.134)	<0.001	4.913 (2.862–8.435)	<0.001
Model 1	2.677 (1.716–4.177)	<0.001	4.080 (2.371–7.022)	<0.001
Model 2	2.671 (1.712–4.169)	<0.001	4.062 (2.360–6.991)	<0.001
Model 3	2.668 (1.710–4.162)	<0.001	4.032 (2.343–6.937)	<0.001
Model 4	2.558 (1.631, 4.012)	<0.001	3.883 (2.244–6.719)	<0.001
Model 5	2.530 (1.611, 3.974)	<0.001	3.859 (2.225–6.691)	<0.001

The low group (GLR <81) was used as a reference. Model 1 = Adjusted for age. Model 2 = Model 1 + sex. Model 3 = Model 2 + multivessel disease. Model 4 = Model 3 + The total length of the stent + the average diameter of the stent + the Killip classification ≥2. Model 5 = Model 4 + eGFR + Fib. GLR, glucose-to-lymphocyte ratio; eGFR, estimated glomerular filtration rate; Fib, fibrinogen; HR, hazard ratio.

### 3.4 Predictive Ability of the GLR in STEMI Patients Who Received Emergency PCI

**Fig. 2.**
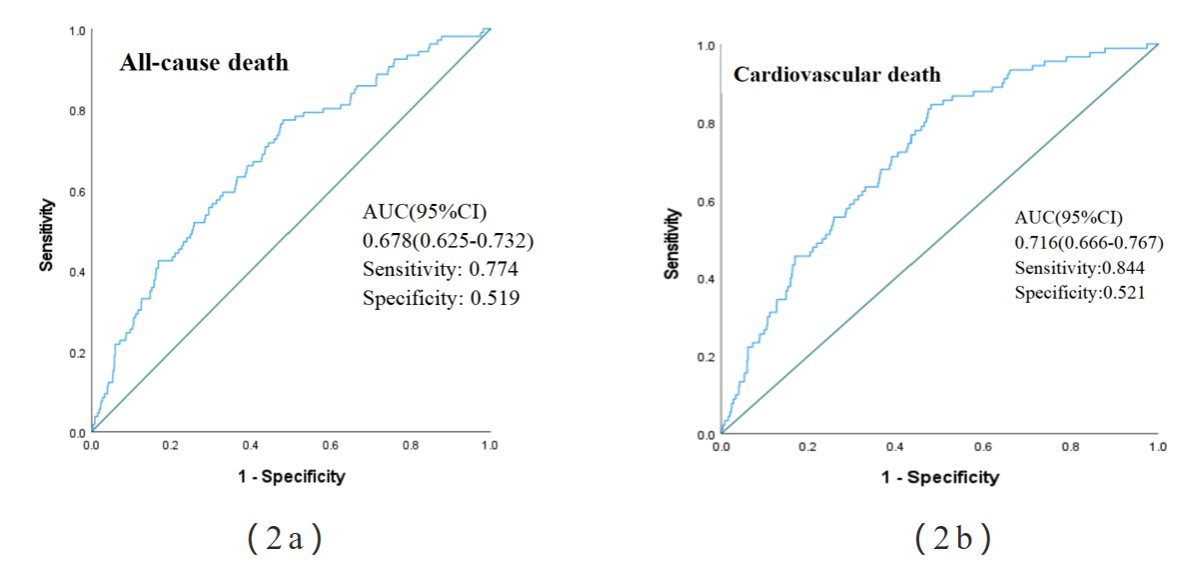
**Time-dependent receiver operating characteristic (ROC) curves for GLR versus all-cause mortality (2a) and cardiovascular mortality (2b)**. AUC, area under the curve; GLR, glucose-to-lymphocyte ratio.

We plotted ROC curves to investigate the prognostic value of the GLR for all-cause death and cardiovascular death in STEMI patients (Fig. 2a,b). The optimal cut-off value of the GLR for predicting all-cause mortality was identified as 79.61 K/µL, demonstrating an area under the curve (AUC) of 0.678 (95% CI: 0.625–0.732, *p* < 0.001), with a sensitivity of 77.4% and a specificity of 51.9%. Comparatively, the optimal cut-off value for predicting cardiovascular mortality was also 79.61 K/µL, yielding a higher AUC of 0.716 (95% CI: 0.666–0.767, *p* < 0.001), along with a sensitivity of 84.4% and a specificity of 52.1%.

**Fig. 3.**
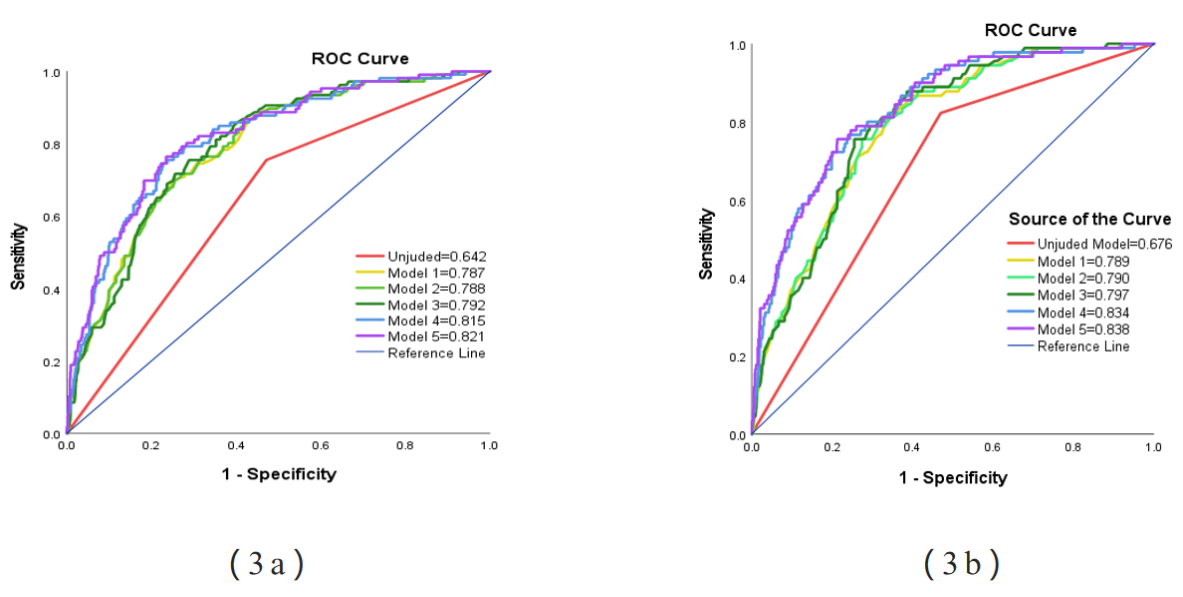
**(3a) (All-cause mortality) and (3b) (cardiovascular mortality): ROC curves according to the predictive value of the six Models for all patients**. Model 1 = adjusted for age. Model 2 = Model 1 + sex. Model 3 = Model 2 + multivessel disease. Model 4 = Model 3 + the total length of the stent + the average diameter of the stent + Killip. Model 5 = Model 4 + eGFR + fibrinogen. ROC curve, receiver operating characteristic curve; eGFR, estimated glomerular filtration rate.

To assess the predictive effectiveness of the models, we plotted the ROC curves of the models, and for all-cause and cardiovascular deaths, the area under the ROC curve of the unadjusted model was 0.642 and 0.676, respectively; in contrast, the area under the ROC curve for Model 1 increased to 0.787 (0.789) when age was added (Fig. 3a,b). Age and sex increased the area under the ROC curve for Model 2 to 0.788 (0.790). Age, gender, and multivessel disease increased the area under the ROC curve for Model 3 to 0.792 (0.797). For model 4, more confounding variables were added, including total stent length, mean stent diameter, and Killip classification, and the area under the ROC curve increased to 0.815 (0.834). Finally, we added eGFR and fibrinogen to Model 5, based on Model 4, and the area under the ROC curve increased to 0.821 (0.838).

## 4. Discussion

This study conducted a retrospective analysis to evaluate the predictive value of the GLR in patients with STEMI who underwent emergency PCI. Our findings demonstrated a significant association between elevated GLR and both all-cause mortality and cardiovascular death. Kaplan‒Meier curves indicated that patients in the high-GLR group experienced a higher incidence of all-cause mortality and cardiovascular events compared to those in the low-GLR group. After adjusting for potential confounding factors, multivariate Cox regression analysis confirmed that an increased GLR is directly linked to a heightened risk of both all-cause and cardiovascular mortality. In Model 5, when a low GLR was used as a reference, all-cause mortality increased 1.530-fold, and cardiovascular death increased 2.859-fold in the high-GLR group. The ROC curve of the model revealed that the constructed model had a good prediction effect. In Model 5, we adjusted for age, sex, multivessel disease status, the total length of the stent, average diameter of the stent, Killip classification, eGFR, and Fib, and the area under the curve for all-cause mortality and cardiovascular mortality reached 0.821 and 0.838, respectively. The GLR is an independent predictor of all-cause and cardiovascular deaths in STEMI patients. Glucose and lymphocytes are readily available, fast, and inexpensive, and the GLR can be a screening tool that helps clinicians identify high-risk populations, assess prognosis early, and conduct closer monitoring and screening to prolong the life of patients.

Hyperglycemia during acute coronary syndrome (ACS) is a common finding and a marker of poor prognosis [18]. In patients who had no previous diabetes diagnosis but were admitted for ACS, new-onset hyperglycemia (NH) was associated with an increased risk of short- and long-term death [19]. Hyperglycemia is associated with myocardial blood flow and energy metabolism alterations, leading to a pro-oxidative and proinflammatory state [20]. In the initial stages of STEMI, the acute glucose response to stress includes hyperglycemia and insulin resistance [21]. Myocardial ischemia leads to an accelerated rate of glycogenolysis and glucose uptake via the glucose transporter-4 receptor to the sarcoplasm [22]. Glucose oxidation requires less oxygen than fatty acid oxidation to maintain adenosine triphosphate (ATP) production. Thus, myocardial energy metabolism is more efficient during glucose oxidation. Due to impaired myocardial glucose uptake in insulin resistance, the ischemic myocardium must utilize trans-fatty acids more than glucose as an energy source. Consequently, in diabetes or insulin resistance, the energy efficiency of the ischemic myocardium is reduced as fatty acid oxidation yields fewer ATP molecules per molecule of oxygen compared to glucose oxidation. The release of fatty acids is further stimulated by the release of catecholamines during stress, which may increase oxygen demand and oxidative stress, leading to myocardial injury and an elevated risk of arrhythmia [23, 24]. High concentrations of free fatty acids (FFAs) in myocardial ischemia increase myocardial oxygen demand and inhibit myocardial activity and contraction [25]. A study has pointed out that stress hyperglycemia in patients with acute myocardial infarction amplifies the inflammatory immune response, which may lead to more extensive cardiac damage and worsen cardiac functional outcomes; meanwhile, an increase in inflammatory immune processes appears to be a possible mechanism linking acute hyperglycemia to poor cardiac prognosis [9]. A study has confirmed hyperglycemia is associated with elevated inflammatory markers and increased infarct size [26]. Some studies have demonstrated that hyperglycemia early in the disease is associated with a poor prognosis for patients with STEMI [21, 27, 28, 29, 30].

Lymphocytes hold a crucial role in the development of inflammation related to STEMI [5]. Lymphocytes are also the most potent predictor of long-term mortality following AMI [7]. The decrease in lymphocyte count in the acute phase of STEMI may be due to increased lymphocyte apoptosis associated with uncontrolled immune system activation [8]. The chemokine fractalkine and its receptor, CX3CR1, are linked to lymphocyte depletion following reperfusion in STEMI. T lymphocytes contribute significantly to myocardial ischemia/reperfusion injury after primary PCI (pPCI), with the fractalkine receptor CX3CR1 playing a pivotal role in this process. Notably, serum fractalkine levels peaked 90 minutes post-reperfusion, coinciding with the lowest observed T cell counts. This depletion in T lymphocytes is a critical factor in the myocardial damage associated with ischemia/reperfusion injury in STEMI patients undergoing pPCI [31, 32]. The study showed that low lymphocyte counts observed within 96 hours of the onset of STEMI were independently associated with an increased risk of recurrent myocardial infarction and increased mortality at the post-discharge follow-up. Patients with lymphocyte counts in the lowest quartile had twice the risk of recurrent myocardial infarction as those in the highest quartile [8]. Cortisol secretion diminishes the number of circulating lymphocytes during myocardial infarction [5]. Animal A study has demonstrated that lymphopenia and immunosuppression may accelerate native atherosclerosis [33]. Lymphocytes play an important role in host immunity by stimulating cytotoxic death and inhibiting cell proliferation [34]. The decrease in lymphocyte counts during the acute phase of STEMI may be due to an increase in lymphocyte apoptosis associated with uncontrolled activation of the immune system, which has been demonstrated in patients with sepsis [35]. Moreover, lymphocyte apoptosis is also present in atherosclerotic lesions. As atherosclerotic plaques develop, lymphocyte apoptosis becomes more frequent and important, contributing to plaque growth, lipid core development, plaque rupturing, and thrombosis [36]. In the cardiovascular domain, lymphopenia has been linked to adverse outcomes across various conditions, including AMI [37], heart failure [38], stable coronary artery disease [39], and unstable angina [40].

To date, research exploring the relationships between the GLR and the risks of all-cause mortality or cardiovascular death in patients with STEMI remains limited. The GLR has emerged as a novel biomarker that integrates lymphocyte counts and blood glucose levels to forecast the prognosis of several diseases. In a pivotal study conducted in 2019, Navarro *et al*. [10] first suggested the predictive capacity of the GLR, identifying it as an independent marker for overall survival and disease-free survival in patients undergoing surgery for T2 gallbladder cancer. A study has revealed that the GLR can independently predict the prognoses of patients with Papillary Thyroid Cancer Patients With Type 2 Diabetes Mellitus [11]. Chen J *et al*. [41] revealed that a higher GLR is an independent prognostic factor for all-cause mortality and cardiovascular disease mortality in patients on peritoneal dialysis. A recent study has shown that elevated preoperative GLR is significantly associated with poorer prognosis in various malignancies, including non-small cell lung cancer, colorectal cancer, breast, gastric, renal, hepatic, esophageal, and melanoma cancers [42]. Liu J and Hu X [17] reported that a higher GLR correlates with an increased risk of in-hospital mortality among AMI patients compared to those with a low GLR. In our study, we observed that STEMI patients treated with emergency PCI and classified within the high-GLR group exhibited a 1.530-fold increased risk of all-cause mortality and a 2.859-fold heightened risk of cardiovascular death post-discharge when contrasted with the low-GLR group. Furthermore, our research indicates that the GLR is an independent predictor for all-cause and cardiovascular mortality. These findings underscore the robust predictive value of the GLR concerning mortality outcomes following emergency PCI in STEMI patients, surpassing the predictive capabilities of traditional markers such as the lymphocyte count or glucose levels.

## 5. Limitations

However, our study also has several limitations. This study is a retrospective observational analysis that may contain some data bias. Second, we primarily investigated patients with STEMI who received emergency PCI; therefore, these results may not be applicable to all individuals with coronary atherosclerotic heart disease.

## 6. Conclusions

Our research shows that the GLR could be a potential inflammatory and metabolic marker and an independent predictor of all-cause death and cardiovascular death in STEMI patients. A high GLR is significantly associated with increased all-cause mortality and cardiovascular mortality. The GLR promises to be an essential predictor of survival for STEMI patients who received emergency PCI treatment. Using the GLR as an indicator, doctors can identify potentially high-risk groups in their clinical work and intervene early to slow disease progression and improve patients’ prognoses.

## Data Availability

Data availability statement All data generated or analyzed during this study are included in this article. Further enquiries can be directed to the corresponding author.

## Abbreviations

STEMI, ST-elevation myocardial infarction; GLR, glucose-to-lymphocyte ratio; PCI, percutaneous coronary intervention; ACS, acute coronary syndrome; AMI, acute myocardial infarction; SBP, systolic blood pressure; DBP, diastolic blood pressure; BMI, body mass index; RCA, right coronary artery; LM, left main coronary artery; LAD, anterior descending branch; LCX, left circumflex artery; ACEI/ARB/ARNI, angiotensin-converting enzyme inhibitors/angiotonin receptor blocker/angiotensin receptor–neprilysin inhibitor; Myo, myoglobin; CK-MB, creatine kinase-muscle/brain isoenzymes; cTnI, cardiac troponin I; WBC, white blood cell; RBC, red blood cell; HB, hemoglobin; PLT, platelet; ALT, alanine aminotransferase; AST, aspartate aminotransferase; ALB, albumin; Cr, serum creatinine; UA, uric acid; eGFR, estimated glomerular filtration rate; TGs, triglycerides; TC, total cholesterol; HDL, high-density lipoprotein; LDL-C, low-density lipoprotein; Fib, fibrinogen; NH: new-onset hyperglycemia.

## Supplementary Material

Supplementary material associated with this article can be found, in the online version, at https://doi.org/10.31083/RCM26065.
