# Supplementary material for: The Glucose-to-Lymphocyte Ratio Predicts All-cause Mortality and Cardiovascular Mortality in ST-Elevation Myocardial Infarction Patients: A Retrospective Study
Source: Rev Cardiovasc Med. 2025 Mar 19;26(3):26065. doi: 10.31083/RCM26065 (PMC11951282; doi:10.31083/RCM26065)

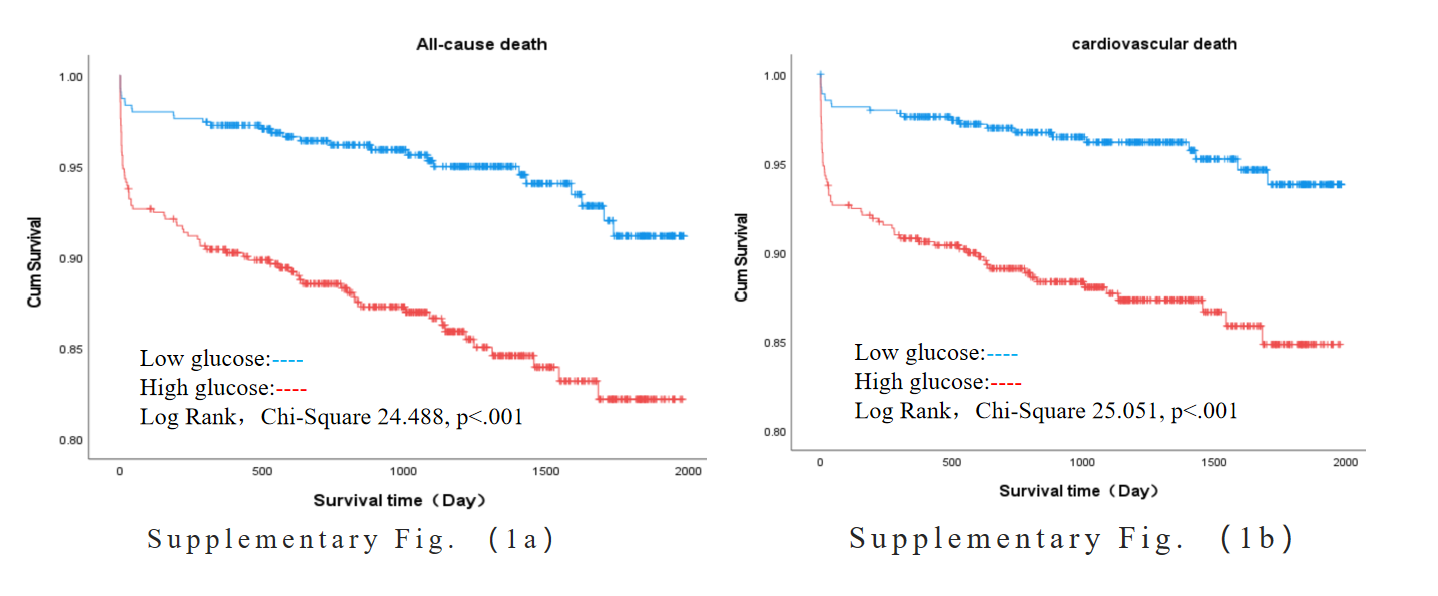


Supplementary Fig. 1a (All cause death) and Fig1b (Cardiovascular death): Kaplan‒Meier survival curves according to the median of the glucose for STEMI patients. Low glucose<6.385, High glucose≥6.385.

Kaplan‒Meier analysis revealed that the cumulative incidence rates of all-cause death (log-rank, χ² 24.488, *p* < 0.001) and cardiovascular death (log-rank, χ² 25.051, *p* < 0.001) were significantly greater in STEMI patients in the high glucose group than in those in the low glucose group. (Supplementary Fig. 1a and Fig1b)


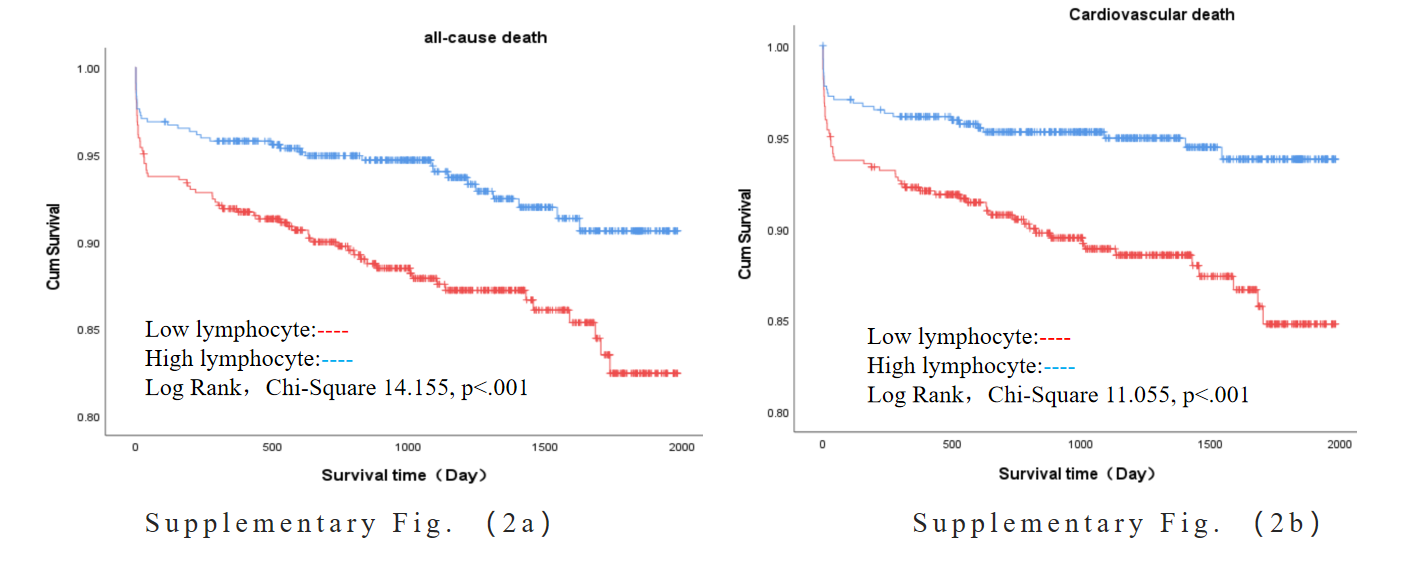


Supplementary Fig. 2a (All cause death) and Fig2b (Cardiovascular death): Kaplan‒Meier survival curves according to the median of the lymphocyte for STEMI patients. Low lymphocytee<1.54, High lymphocyte≥1.54.

Kaplan‒Meier analysis revealed that the cumulative incidence rates of all-cause death (log-rank, χ² 14.155, *p* < 0.001) and cardiovascular death (log-rank, χ² 11.055, *p* < 0.001) were significantly greater in STEMI patients in the low lymphocyte group than in those in the high lymphocyte group. (Supplementary Fig. 2a and Fig2b)

If necessary, Attachment: Flowchart


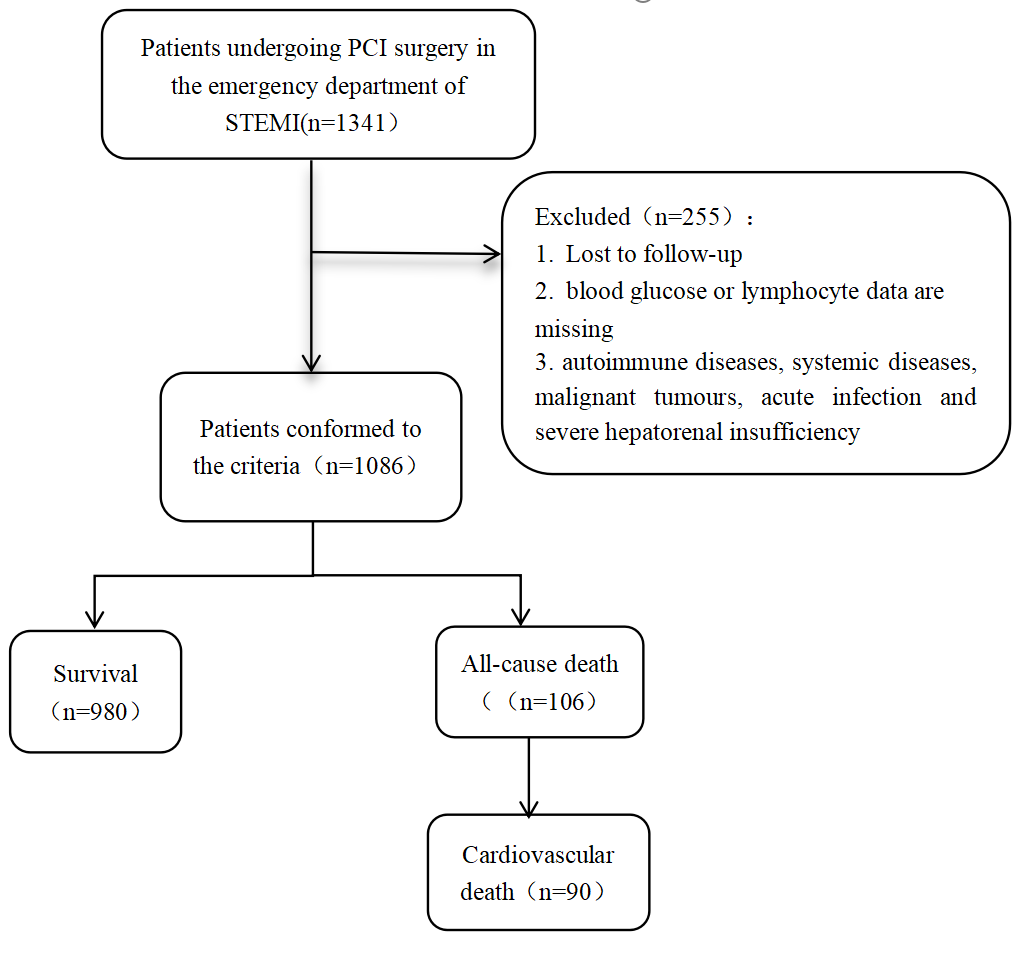

Supplement: Supplementary file 1 [file 2153-8174-26-3-26065-s1.docx]
